# Surgery Versus ATMPs: An Example From Ophthalmology

**DOI:** 10.3389/fbioe.2020.00440

**Published:** 2020-06-10

**Authors:** Federica M. Magrelli, Alessia Merra, Graziella Pellegrini

**Affiliations:** ^1^Holostem Terapie Avanzate S.r.l., Modena, Italy; ^2^Stefano Ferrari Regenerative Medicine Center, University of Modena and Reggio Emilia, Modena, Italy

**Keywords:** ATMP, surgery, ophthalmology, cost of treatment, public–private health care

## Abstract

Advanced therapy medicinal products (ATMPs) are the new frontier of medicine. Advanced therapy medicinal products are set out to satisfy unmet medical needs and provide new innovative, cutting-edge therapies for serious or life-threatening diseases, thus providing new therapeutic options for people with few or no possibility of treatment. They are divided into four groups including gene therapy medicinal products, cell-based therapy medicinal products, tissue-engineered products, and combined ATMPs, which in Europe refer to products that incorporate one or more medical devices with any of the previously mentioned ATMPs as part of the advanced medicine product (AIFA, 2017; Ten Ham et al., 2018). Advanced therapy medicinal products can potentially have long-term benefits, thus bringing a long-lasting positive impact on patient health. Advanced therapy medicinal product therapies are often administered just once or twice, which gives patients the possibility to heal quickly compared to traditional therapies. They also provide a long-term saving opportunity, both in terms of costs of treatments and procedures that are no longer necessary and in terms of quality of life and productivity. The resolution of the patient’s illness has a monetary impact on the patient, the patient’s caretakers, and especially on the society (Alliance for Regenerative Medicine, 2019). The aim of this paper was to provide an overview on the use of ATMPs approved in Europe, with a focus on blindness and visual impairment and the related economic burden. In this case study, the effective cost of a blind patient in different European countries was compared after treatment with ATMPs or traditional therapies, focusing on visual impairment caused by corneal opacity. Our evaluation includes an overview of the global economic impact of the two types of therapies on the society. We estimated direct healthcare costs, direct non-healthcare costs, and labor productivity losses, to include costs on healthcare, services, patients, their families and for the society in general. We could conclude that the costs of the two therapeutic approaches are comparable.

## Introduction

Advanced therapy medicinal products (ATMPs) have unique attributes which differentiate them from standard pharmaceuticals and biologics. Indeed, ATMPs have curative potential as they address underlying genetic or cellular mechanisms of disease, which means that they can have a dramatic and long-lasting positive impact on health. They act through multiple mechanisms and on different cellular targets.

ATMPs are often administered just once or a handful of times within a short period. However, as they are typically paid as a one-time treatment, they have a high up-front cost. They are complex products and thus, have also difficult manufacturing processes, often requiring highly specialized manufacturing equipment, processes, and skills. Many cell-based gene therapies, for example CAR-T therapy used to treat some blood cancers, are individually manufactured for each patient: cells are collected from the patient’s blood using a process called apheresis, they are modified and expanded in the laboratory, and then re-infused into the patient several hours later. These processes are usually carried out by trained individuals in specialized centers.

Overall, ATMPs can also have a positive impact on patient quality of life, caregivers and on the whole society. In fact, the use of ATMPs decreases hospitalization, avoids continuous drug administration, and reduces nursing. Notably, this approach speeds up the patient productivity, enabling quick return to work with no burden on the society.

Thus, ATMPs appear to have the extraordinary potential to offer durable, life-changing solutions for the society. These highly complex treatments rely on current surgical practice and could not prescind from it, but differ from traditional medicines, both in terms of how they are made/administered and by the type of benefits they may provide.

In particular, the 3.3% of total number of ATMP clinical trials worldwide are in the field of ophthalmology (Alliance for Regenerative Medicine, 2019). Globally, it is estimated that there are about 2.2 billion people with vision impairment or blindness and at least 1 billion people with a form of vision impairment that could have been prevented or has yet to be addressed (World Health Organization, 2019).

## ATMPs Approved in Europe

Advanced therapy medicinal products can be classified into three main types:

• **Gene therapy medicinal products:** consist of a vector or delivery formulation containing genes that lead to a therapeutic, prophylactic, or diagnostic effect. The genetic construct is engineered to express a specific transgene. The ‘recombinant’ genes are inserted into the body and by using such gene therapy constructs, *in vivo* genetic regulation or genetic modification of somatic cells can be achieved. A recombinant gene is a stretch of DNA that is created in the laboratory, bringing together DNA from different sources (European Medicines Agency, 2018).
• **Somatic-cell therapy medicinal products:** consist of cells or tissues that have been subjected to substantial manipulation, or that are not intended to be used for the same essential function(s) in the recipient body. They can be used to cure, diagnose, or prevent diseases.
• **Tissue-engineered products:** these contain engineered cells or tissues that have been modified so they can be administered with the aim of regenerating, repairing, or replacing human tissue.

In addition, there are ATMPs that consist of one of the first three categories combined with one or more medical devices as an integral part of the product, which are referred to as combined ATMPs (European Medicines Agency, 2017).

Up to June 2019, a total of 14 ATMPs have been granted marketing authorization in Europe: seven gene therapies, four cell therapies, and three tissue engineered products. However, four ATMPs have been withdrawn from the market because they did not obtain any reimbursement (Table 1; Ten Ham et al., 2018; Alliance for Regenerative Medicine, 2019).

**TABLE 1:** ATMPs approved in Europe (Alliance for Regenerative Medicine, 2019).

**Drug name**	**Developer**	**Indication**	**Approval date (EU)**	**Status**	**Therapy**
Chondrocelect^®^	TiGenix	To repair a cartilage defect of the knee	October 2009	X 01-2017	CT
Glybera^®^	uniQure	For lipoprotein lipase deficiency (LPLD)	October 2012	X 10-2017	GT
MACI^®^	Vericel	To repair a cartilage defect of the knee	June 2013	X 09-2014	T-B T
Provenge^®^	Dendreon	To treat advanced prostate cancer in men in whom chemotherapy is not yet clinically indicated	September 2013	X 05-2015	CT
Holoclar^®^	Holostem	In adult patients with moderate-to-severe limbal stem-cell deficiency caused by burns, including chemical burns to the eyes.	February 2015	✓	T-B T
Imlygic^®^	Amgen	For regionally or distantly metastatic unresectable melanoma	December 2015	✓	GT
Strimvelis^®^	GSK	Adenosine deaminase (ADA)−deficient severe combined immunodeficiency (SCID)	May 2016	✓	GT
Zalmoxis^®^	MolMed	Add-on treatment for HSCT of adult patients with high-risk haematological malignancies	August 2016	✓	CT
Spherox^®^	CO.DON	To repair a cartilage defect of the knee	July 2017	✓	T-B T
Alofisel^®^	TiGenix	To treat complex anal fistulas in adults with Crohn’s disease	March 2018	✓	CT
Kymriah^®^	Novartis	Certain types of acute lymphoblastic leukemia in people up to 25 years old and in certain adult patients with large B-cell lymphoma	August 2018	✓	GT
Yescarta^®^	Gilead	CAR T therapy for adults living with certain types of non-Hodgkin lymphoma who have failed at least 2 other kinds of treatment.	August 2018	✓	GT
LUXTURNA^®^	Novartis	To treat an inherited retinal disease, indicated for children and adults with vision loss caused by mutations in both copies of the RPE65 gene and enough viable retinal cells	November 2018	✓	GT
Zynteglo^®^	BlueBird Bio	To treat a blood disorder known as beta thalassemia in patients 12 years and older who require regular blood transfusions	June 2019	✓	GT

*GT, gene therapy; CT, cell therapy; T-B T, tissue based therapy; ✓, authorized; X, withdrawn.*

Among the 10 approved ATMPs, two focus on eye diseases. More specifically, they have been developed to cure blindness or visual impairment. In 2014, the Committee for Advanced Therapies (CAT) recommended Holoclar^®^, the first ATMP ensuring a specific number of stem cells, for the treatment of moderate and severe Limbal Stem Cell Deficiency (LSCD). In February 2015, Holoclar^®^ received conditional approval by the European Medicines Agency (EMA) for the use in the European Union (EU; European Medicines Agency, 2015).

The second was Luxturna^®^, approved in 2018 as the first gene therapy to restore vision in people with rare inherited retinal disease, caused by mutations in the *RPE65* gene. This therapy can be provided to patients with enough residual cells in the retina. Ten years of evidence of its safety were proposed and its use has been studied in patients with ages ranging between 4 and 44 years old.

However, being approved in November 2018, Luxturna^®^ has not enough data available to assess the product’s impact. It is still too early for a comparison of outcomes of patients treated with Luxturna^®^ versus traditional therapies, after authorization (Luxturna, 2018).

Notably, the comparison of the social impacts of different types of therapies can be done when: (i) adequate time from approval is available to evaluate economic consequences, (ii) significant follow-up of patient outcomes is collected with the approved technique, and (iii) routine treatments are available as comparator of ATMP effects.

For example Strimvelis^®^, approved in 2016, has long term follow-up data, but no comparators for economic analysis are available.

As a consequence, we analyzed the use of the first CLET (Cultured limbal epithelial transplantation: Holoclar^®^) for the treatment of blindness and visual impairment. This product was launched in 2015, therefore, extensive information are available on more than 100 patient outcomes with several years of follow up. In addition, alternative approaches are available as comparators, as described below.

## Global Data: Blindness and Visual Impairment

Eye health has profound and wide-spread implications in many aspects of life, health, sustainable development, and economy.

Worldwide, visual impairment leads to a considerable economic burden for both affected and non-affected people. The estimated number of people with sight damage is more than 217 million, of which 47 million have severe damage and 170 million have moderate damage, while the number of people who are blind is estimated to be 36 million (Table 2; Bourne et al., 2017; The Lancet Global Health Commission, 2019).

**TABLE 2:** Global data on visual impairment and blindness in 2015 (Bourne et al., 2017).

	**World population (million)**	**Blind (million)**	**Moderate-to-severe VI (million)**	**Mild VI (million)**
*TOTAL*	7330	36	217	188.5
*MEN*	3700	15.87	97.76	87.11
*WOMEN*	3630	20.14	118.85	101.44

*VI, visual impairment.*

Globally the major causes of visual impairment are uncorrected refractive errors (43%) and cataracts (33%). Other causes are glaucoma (2%); and age-related macular degeneration (AMD), trachoma, diabetic retinopathy, and corneal opacities (1%). Furthermore, a large proportion of cases (18%) have an undetermined cause.

**FIGURE 1:**
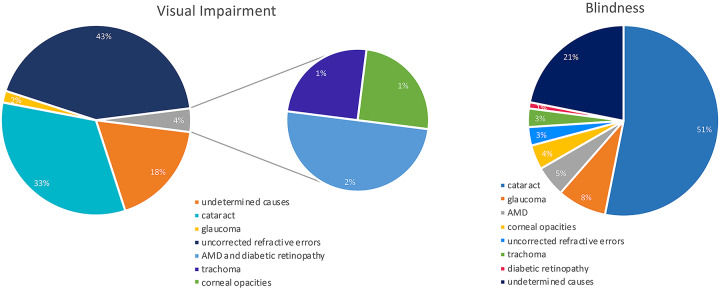
Causes of global blindness and global moderate-to-severe visual impairment in 2010 (Pascolini and Mariotti, 2012).

Meanwhile, the main causes of blindness are cataracts (51%), glaucoma (8%), AMD (5%), corneal opacities (4%), uncorrected refractive errors and trachoma (3%), and diabetic retinopathy (1%), while 21% of cases have undetermined causes (Figure 1; Pascolini and Mariotti, 2012).

## Socio-Economic Impact of Visual Impairment and Blindness: Examples From European Countries

**FIGURE 2:**
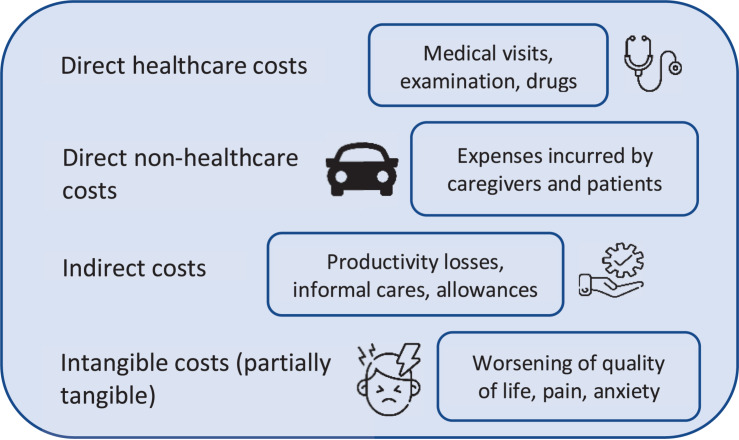
Schematization of total costs for a blind and visually impaired patient.

Visual impairment and blindness have considerable socio-economic consequences attributable to the following:

- ***Direct healthcare costs*** incurred within the healthcare system by the government and/or other payers. These include, for example, general ophthalmic services, hospitalizations, treatment, expenditure associated with injurious falls due to visual impairment and blindness, and rehabilitation.
- ***Direct non-healthcare costs*** caused by the illness but not imputable to medical treatment: e.g., home improvements (e.g., ramps, door-opening devices, handlebars and tactile assistance systems), technical assistance such as sticks, guide dogs or computer interface, mobility, home care.
- ***Indirect costs*** include the economic impacts of this condition outside the healthcare system and on the wider society. These include:
  - *Reduced productivity* of the patients and their caregivers due to absenteeism, limited efficiency at work (presenteeism), part-time employment or loss of work,
  - *Informal cares* (family or social care),
  - *Social security costs* (invalidity pensions or accompanying allowance, financial support for income, residence, or benefits).
- ***Intangible costs*** reflecting the burden of the disease in terms of worsening of the patient’s quality of life due to, for example, pain and other aspects such as the stress felt by the caregiver. Indeed, these can be tangible to some extent, such as in cases of costs related to depression, anxiety and further excess of morbidity. These can be traced back to other types of direct healthcare costs that can be easily calculated (Javitt et al., 2007; Figure 2).

Among direct non-healthcare costs, those dedicated to home improvements are not considered in a first analysis, but they have a consistent weight on total costs, as analyzed below.

Home adaptation-costs, reported from Italy, include kitchen and bathroom adjustments such as tactile assistance systems; cost €1,003.30 and €5,994.3, respectively. Similarly, in France, it is reported that the cost for a stair lift for a single patient is €6,000, while in Germany the cost of door-opening devices is €1,900 per unit (Lafuma et al., 2006).

Studies on cost of illness are a pivotal measure in healthcare, to assess the economic burden of a disease on the society. These studies support the quantification of the “hidden” costs of illness and so help to reveal the true disease-related charges. This is important because costs have a key role in public policy making and could help decision makers to prioritize medical costs, including research.

In fact, doing a cost-benefit analysis (CBA), the inclusion of marginal cost in the evaluation is emphasized, as it should not be a “on/off” decision, but rather a “more/less” decision.

An economic calculation may reveal, despite an apparent level of spending, that the additional (marginal) costs can change the final result of the cost evaluation (Zweifel and Telser, 2009).

Investments on ATMP that could cure patients with visual impairment, will lead to a healthier population, which in turn, could result in a more affordable medical budget for governments, a healthier tax-paying workforce, and lower productivity losses, improving the wellbeing and quality of life of patients and their caregivers.

**FIGURE 3:**
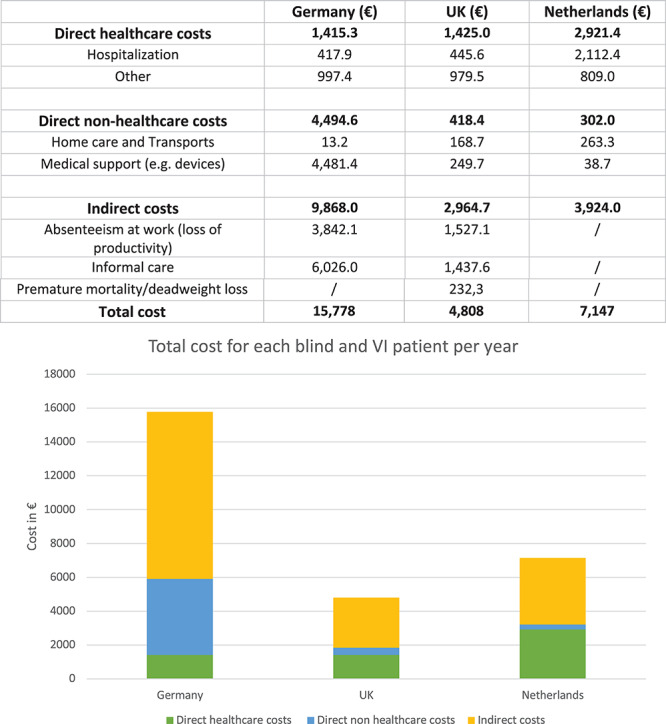
In this table and figure are represented the direct healthcare costs, the direct non-healthcare costs and the indirect costs for some European Countries per unit (for each blind and visually impaired patient) (Pezzullo et al., 2018; Schakel et al., 2018; Chuvarayan et al., 2019). VI, visually impaired. Numerical data for UK have been converted from GBP to Euro.

Here, we analyzed the annual costs of blindness and visual impairment reported in three western countries (from EU) in order to compare direct healthcare costs, direct non-healthcare costs, and indirect costs (Figure 3). Indeed, three studies were proposed by Netherland (Schakel et al., 2018), United Kingdom (Pezzullo et al., 2018), and Germany (Chuvarayan et al., 2019) for a cost-benefit analysis; this CBA was based on a previously made contingent valuation (CV), used to estimate economic values for all kinds of services. In the selected countries, the global cost for all visual impaired and blind individuals was divided by the number of the total patients in each Country, in the specific year (Zweifel and Telser, 2009). In particular, results revealed that the direct healthcare costs of blind and visual impaired individuals represented just a small percentage of the total cost of this disability. Instead, the largest percentage of the costs, was due to productivity loss, social security costs, and informal support/care by caregivers. These results highlight the crucial role that indirect costs, usually not considered, play in the total cost of illness.

## Economic Benefit of ATMPs Compared to Traditional Therapies: Focus on Treatment of Limbal Stem Cell Deficiency

The aim of the treatment for LSCD is to restore the surface of the eye, achieve corneal clarity and improve visual acuity in monolateral or partial bilateral blindness. Current treatment practices usually start with supportive care treatments such as lubrication, autologous serum eye drops, antibiotics, anti-inflammatory drugs, and therapeutic soft or scleral contact lenses. Conservative surgery such as corneal scraping may also be offered before attempting limbal stem cell transplantation. The latter includes several types of invasive surgical options, the aim of which is to transplant stem cells to the affected eye. The surgical options differ in terms of where the cells come from and how they are transferred, specifically the following:

• Conjunctival limbal autograft (CLAu), in which stem cells are taken from the contralateral uninjured limbal tissue from the patient’s healthy eye.
• Conjunctival limbal allograft (CLAL), in which stem cells are taken from a living, related donor or dead donor and transplanted into the diseased eye of the recipient.
• Keratolimbal allograft (KLAL), transplants the entire limbus from a dead donor using the corneoscleral carrier to deliver a large number of stem cells to the recipient.
• Simple limbal epithelial transplantation (SLET), reduces the tissue withdrawal of CLAu, but it can treat milder severity (superficial lesions) than CLET. In the SLET procedure, re-epithelialization is slower than in some of the other therapies (it takes about 5–6 weeks) (Sangwan et al., 2012; Swapna et al., 2019).
• Cultured limbal epithelial transplantation (CLET) procedure (Pellegrini et al., 1997) has some advantages compared to CLAu, for example it starts from a smaller amount of limbal tissue (1–2 mm^2^), minimizing the risk of injury to the healthy eye. In addition, CLET does not require lifelong immunosuppression and in cases of failure, the treatment can be repeated multiple times.

Due to the evidence of its safety and efficacy, one specific CLET technique, under the name Holoclar^®^, has been conditionally approved in 2015 by the EMA as the first stem cell−based therapy (European Medicines Agency, 2015).

Some non ATMP-treatments can have disadvantages. For example, CLAu requires a large amount of donor tissue from the healthy eye (equivalent to around 40% of the available donor cornea). This increases the risk of damage to the donor eye and the treatment cannot be repeated in case of failure. The CLAu technique leads complete corneal epithelialization from day 18 up to several weeks after surgery (Kheirkhah et al., 2008).

In contrast, the use of the approved cultured stem cell therapy (Holoclar^®^) has several advantages, such as the absence of immunological rejection (autologous cells do not require immunosuppression), the use of a small limbal biopsy (1–2 mm^2^), and the standardization of each preparation of the product made individually from the donor’s cells for a single treatment. It is important to note that treatments can be repeated multiple times if both eyes need to be cured (European Medicines Agency, 2015; NICE, 2017a).

Thus, the cost of each traditional therapy could appear lower than the cost of an advanced therapy (see Tables 3.1–3.3), however, a more global evaluation of ATMPs leads to a different conclusion. Advanced therapy medicinal products can reduce hospital stay, medical evaluations, additional therapies, nursing costs, and finally, both direct non-healthcare and indirect costs. In fact, cost evaluation regarding cases of vision loss or blindness requires inputs to assist decision makers (e.g., surgeons, patients, payers), to calculate the cost effectiveness of different treatments as a whole and prioritize health expenditure for the society.

**TABLE 3.1:** Summarizes an example of costs for different LSCD treatments.

	**LSCD Costs up to Surgery (€)**	
CLAu		€ 21,893
Lr-CLAL		€ 65,479
KLAL		€ 77,393
SLET		€ 21,000*
BSC		€ 88,377
Holoclar		€ 93,907

CLAu, limbal conjunctival autograft; Ir-CLAL, conjunctival limbal allograft tissue from living relatives; KLAL, keratolimbal allograft; BSC, best supportive care, SLET, simple limbal epithelial transplantation. Data have been converted from GBP to Euro from NICE (2017a). *Data estimated by Table 2 (Sangwan et al., 2012) that consider that SLET has the same cost as CLAu.

**TABLE 3.2:** Comparison between therapies – qualitative analysis.

	**CLET**	
	**(Holoclar^®^ + surgery)**	**SLET**
**Costs to add to each surgery**		
Hospitalization after surgery	1 day	Variable
**Drugs/Medications needed**		
Therapeutic contact lens	Not required	Yes
Amniotic membrane	Not required	Yes
Antibiotic eye drops	+	++
Steroid eye drops	+	++
Artificial tears	+	+
Outpatient appointments (first year)	At least 6 appointments	At least 9 appointments
**Other costs to add to each therapy**		
Home treatment	Self-medication	Medications At least 5 weeks up to complete re-epithelialization
Cost up to complete epithelialization	3–7 days	5–6 weeks
Invalidity/productivity loss	Days	Months
Pharmacovigilance on adverse event	Yes	Absent
Reproducibility of results	Highly standardized GMP setting	Not standardized setting
Proven inter-hospital consistency	Yes	No

*This Table lists different treatments included in each therapy. The list of drugs and medications was taken from Basu et al. (2016) for SLET and AIFA (2019) for Holoclar. + used during the therapy, + ⁣ + used in higher amount than the comparator.*

**TABLE 3.3:** Comparison between therapies – quantitative analysis.

	**CLET (Holoclar^®^)**	**SLET**
Up-front cost of therapy	€ 93,907 (cost of surgery)	€ 21,000
**Long-term healthcare costs**		
Long-term stability**	23.4% failures up to 10 years (based on a proven follow-up)	24.8% failures up to 4 years (based on a proven 4 years follow-up) + 6 years hypothetical stability (best case) Or 6 years potential 100% failure (worst case)
Total potential cost of failures ^§^ in 10 years (follow-up)	€ 206,802	€ 220,943–€ 618,639
Total potential partial cost including surgery	€ 300,709	€ 241,943–€ 639,639

In this table, the percentage of failure has been calculated from Rama et al. (2010) for Holoclar^®^ and Basu et al. (2016) for SLET. **Failure rate and long-term stability were calculated on reported successful outcomes and proven 10 years follow-up for Holoclar^®^ (Rama et al., 2010), and 4 years follow-up reported for SLET (Basu et al., 2016). The following 6 years (required to compare the data with ATMP) of SLET have been considered as a range: from a best case scenario as stable percentage of failure (24.8%) (Basu et al., 2016) to the worst case of late failure (100%). **^§^** The cost of failure was based on expenses for Best Supportive Care (BSC in Table 3.1).

Procedure standardization, at the production and clinical application level, implies a clear definition of reproducibility on raw materials, production, clinical protocol, training of surgeons, long-term follow-up, and monitoring of adverse events, as requested by regulatory authorities.

Standardization has an impact on cost evaluation of the procedure. The absence of these guarantees results in highly variable results (potentially 0–100%), unknown long-term efficacy, and absence of pharmacovigilance on adverse events. Altogether, these missing points produce uncontrolled long-term increase/variability of costs (of the procedure) as calculated in Table 3.3.

**FIGURE 4:**
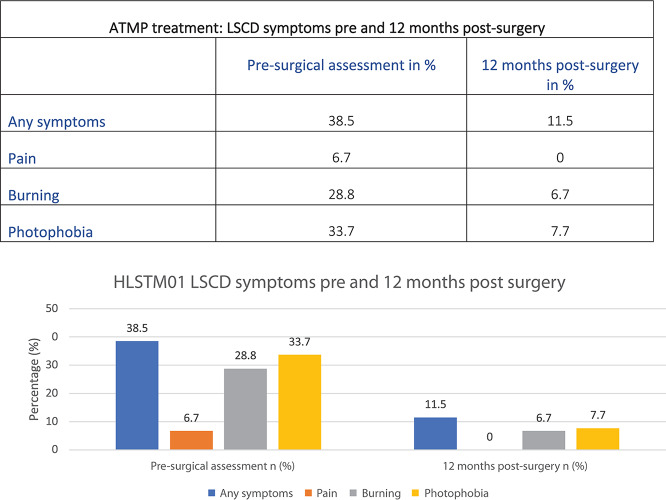
Data from the first Holoclar clinical trial (HLSTM01) based on 97 patients (NICE, 2017a).

Another advantage of the ATMP is that it uses new autologous healthy tissue grown in the laboratory that can be implanted onto the damaged cornea, healing the receiving eye in a few days with a rapid decrease in the patient’s symptoms (Figure 4).

Results in Figure 4 could be explained by the minimally invasive surgery required. Patients are usually able to go back home in one or a few days, compared to traditional surgeries requiring longer healing time.

Current clinical practices mainly rely on day surgeries, with a decrease on in-hospital patient average length of stay (days) in EU countries over the past few years, although the pace of diffusion has varied widely across countries.

**FIGURE 5:**
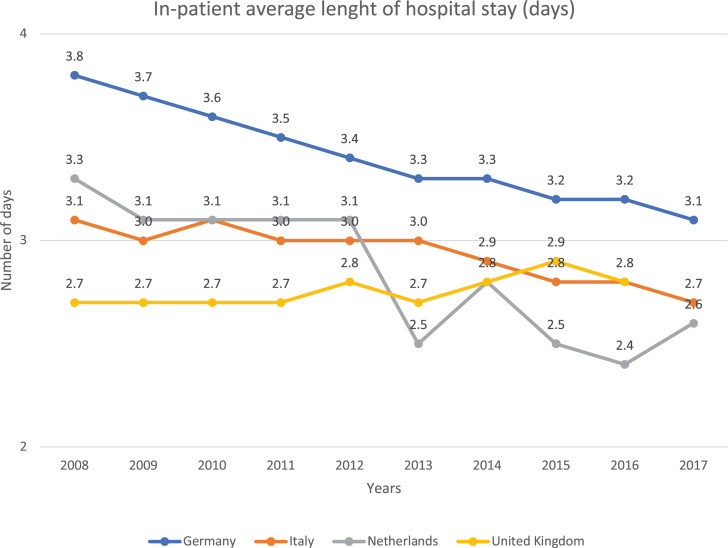
In-patient average length of hospital stay (days) in different EU countries. This figure shows the number of days a single patient stays in hospital. It shows that the number of days in the hospital decreases with the years. These data refer to “diseases of the eye and adnexa” EUROSTAT category (Eurostat Database, 2016).

Indeed, Figure 5 shows that hospitalization has been reduced over the past 10 years, highlighting a clear tendency to decrease this cost for the society.

Therapies not following this trend, require an extension of the recovery and associated medical evaluations; they create cost-related problems and, above all, organizational problems to the entire healthcare system.

It is worth noting that patients with a less severe condition and an acceptable quality of life are less likely to suffer co-morbidities or adverse events requiring further, potentially expensive, therapies and support. Different studies have showed that visually impaired patients suffer from increased levels of depression, psychological stress, anxiety, and mental fatigue. It is estimated that depression occurs in patients with visual impairment more often (about 17%) than in patients with no vision damage (Van der Aa et al., 2016). Thus, depression is an additional cost for the society that could be avoided with timely patient treatment and effective sight recovery. For example, in Netherlands, psychological rehabilitation for each visually impaired patient costs about €432.6 per year (Schakel et al., 2018). This continuative cost, for both blind or visually impaired patients, for several years is a burden for the society and cannot be ignored.

## Conclusion and Future Perspectives

A careful scientific and economic evaluation of the additional costs of each therapy should drive the selection of affordable medical solutions. As the number of ATMPs is increasing, the high prices associated with them have become the topic of many debates. Here, we have focused on sight recovery as it is a critical issue worldwide.

Beyond the value of ATMPs for healthcare and considering the total cost for a single patient, it is important to consider healthcare-related costs as well as non-healthcare and indirect costs to perform an appropriate evaluation. It is necessary to have a holistic view on expenses for the government, especially in the case of therapies with high up-front costs.

Hospitalization, home care, medical evaluations and adverse events are the most relevant costs for a blind or visual impaired patient. However, costs are not only related to the length of stay in hospital, but also to the hospital’s logistic expenses, to the cost of physicians and nurses that take care of patients for months and to other expenditures. Such costs are widely reduced with the new innovative therapies.

Nevertheless, it is also important to consider the global experience of the patient and the possible correlated illnesses such as infections, pain and depression caused by prolonged and non-resolutive therapies. Patients often undergo long-term treatments with drugs in order to reduce symptoms but without any sight restoration. They live with pain and related depression and are not productive, representing often a burden for the society (NICE, 2017b).

Supportive care or surgical approaches can appear, at first, as cheaper choices but, afterward, they are often less effective than new therapies. These last ones potentially bring more significant benefits, especially in the long-term, not only for the success of the treatment but also for the caretakers, families, and for the society as a whole.

Therefore, the main aim of each therapy and treatment is patient’s healthcare, its satisfaction and self-sufficiency, all in the frame of economic sustainability.

Finally, the costs of ATMPs include GMP production costs. The apparently high up-front costs of ATMPs are compensated by the high levels of therapy standardization and safety, ensuring a cost/benefit ratio. Concerning R&D, its related costs are compensated by the usefulness of R&D in assessing public policies and stimulating drug development and innovation.

Overall, our analysis highlights that, globally, there is not increase in the costs of ATMPs versus surgery, due to their guarantee of success and short duration.

## Author Contributions

FM and AM provided the acquisition. GP, FM, and AM analysis and interpretation of data for the work, drafted the work and revised it and approved for the publication of the content. All authors are accountable for all aspects of the work.

## Conflict of Interest

FM and AM have a training employment by the company Holostem Terapie Avanzate, author GP is a member of the Board of Directors and R&D Director of Holostem Terapie Avanzate.
